# The effect of high approach-motivated positive affect on selective attention under high perceptual load

**DOI:** 10.3389/fnhum.2025.1628818

**Published:** 2025-08-13

**Authors:** Fang Liu, Qin Zhang

**Affiliations:** ^1^Key Research Base of Humanities and Social Sciences of the Ministry of Education, Academy of Psychology and Behavior, Tianjin Normal University, Tianjin, China; ^2^Faculty of Psychology, Tianjin Normal University, Tianjin, China; ^3^Learning and Cognition Key Laboratory of Beijing, School of Psychology, Capital Normal University, Beijing, China

**Keywords:** positive affect, approach motivation, perceptual load, selective attention, ERP

## Abstract

**Introduction:**

Selective attention is a crucial mechanism that enables humans to navigate complex environments and accomplish targeted tasks, garnering significant interest from researchers. Numerous studies have found that selective attention can be influenced by emotions; however, previous research has primarily focused on the effects of valence and arousal, neglecting the role of motivation, another dimension of emotion. Additionally, it remains unclear whether emotional motivation’s influence on selective attention differs across various levels of perceptual load.

**Methods:**

This study employed a modified perceptual load Flanker task, using behavioral measures and event-related potentials (ERPs) technique to investigate how the intensity of approach-motivated positive affect influences selective attention under different levels of perceptual load. In each trial, participants were first shown pictures of food or scenes to induce high or low approach-motivated positive affect, followed by a modified perceptual load Flanker task where a searchable array of letters was arranged in a virtual circle around a central fixation point, with an interference letter presented on either the left or right periphery. The searchable array included one target letter and either five identical (low perceptual load) or different irrelevant letters (high perceptual load). Participants were required to identify the target letter and respond with a button press.

**Results:**

The findings revealed that under conditions of high perceptual load, participants with low approach-motivated positive affect exhibited slower reaction times than those with high approach-motivated positive affect. Meanwhile, the ERP results indicated that under high perceptual load, low approach-motivated positive affect induced a greater N1 in the parieto-occipital region compared to high approach-motivated positive affect. Additionally, high approach-motivated positive affect evoked a greater N2 in the frontal region and a smaller P3 in the parietal region compared to low approach-motivated positive affect and neutral affect.

**Discussion:**

These results demonstrate the inhibitory effect of low approach-motivated positive affect and the enhancing effect of high approach-motivated positive affect on performance in the high perceptual load Flanker task.

## 1 Introduction

Selective attention is the process by which individuals concentrate on specific stimuli within their environment. Given the limited capacity for information processing, people prioritize certain information based on their immediate needs or objectives, while other details are often overlooked. Selective attention comprises two mechanisms: an early sensory input selection mechanism that inhibits the perception of task-irrelevant distractors, and a late response selection mechanism that mitigates the distractions caused by interfering stimuli, particularly when these distractors have been perceived (Lavie et al., 2004). Selective attention could be influenced by many factors. In the first place, the perceptual load theory of selective attention (Lavie, 1995; Lavie et al., 2004; Lavie, 2010) posits that the perceptual load of a task can influence selective attention. Perceptual load refers to the total amount of content that working memory must process, which equates to the cognitive resources consumed to process the current task. If the current perceptual load is high (for example, detecting a target among multiple irrelevant stimuli), and resources are exhausted, attention can only select objects or information related to the current task. There are no extra resources available to process information unrelated to the task, resulting in an early sensory input selection by attention. Conversely, if the current perceptual load is low (for example, detecting a target among identical irrelevant stimuli), excess resources will automatically spread to other irrelevant objects or information. Processing this irrelevant information will interfere with the execution of the current task, resulting in a late attention selection (Lavie and De Fockert, 2003; Lavie et al., 2014). Under high perceptual load, due to insufficient cognitive resources, interference stimuli are not processed, and attention selection reflects the perceptual selection mechanism. However, under low perceptual load, with sufficient cognitive resources to process interference stimuli, cognitive control is required to enhance the response to the target stimulus, and attention selection involves an active process. That is, the interference item is activated during the early stages of attention by the remaining attentional resources, and then it is suppressed during the later stages by other residual attentional resources (Lavie, 2005; Lavie and Cox, 1997; Cartwright-Finch and Lavie, 2007; Forster and Lavie, 2008a,b; Tan et al., 2015).

Moreover, selective attention can also be influenced by an individual’s emotions and motivation. In this context, most previous studies on emotions have primarily focused on valence. Positive emotions tend to enhance attention span and cognitive flexibility, while negative emotions often have an inhibitory effect (Fredrickson, 2001; Hu et al., 2012). However, Gable and Harmon-Jones (2008, 2010a,2010b) proposed the motivation model of emotion, which incorporates motivation as a key dimension of emotion, further dividing it into two aspects: intensity and direction. Intensity can be characterized as either high or low, while direction can be categorized as either approach or avoidance. Approach motivation refers to the drive or behavioral inclination to move toward certain things or situations, whereas avoidance motivation refers to the tendency to move away from specific things or situations. Researchers have investigated the influence of positive affect on attention by manipulating the motivational dimension of emotion. An event-related potential (ERP) study (Liu et al., 2016) examined the influence of high and low approach-motivated positive affect on selective attention using a Flanker task with added detection stimuli. This study found that the intensity of emotional motivation influences both the early and late stage of attention processing. High approach-motivated positive affect narrows the scope of attention during the visual input stage and enhances interference suppression compared to low approach-motivated positive affect.

Prior research has established that selective attention involves both top-down and bottom-up processes (Jefferies et al., 2019). It is influenced by emotional background in a top-down manner and by cognitive load in a bottom-up manner, respectively. However, an intriguing but unresolved question is how selective attention would be affected when perceptual load and emotional background are simultaneously manipulated? What would occur when the influence of top-down emotional background is consistent or inconsistent with the influence of bottom-up task demands? At which stage of attention processing does the combined effect of perceptual load and emotional motivation emerge? These questions highlight important gaps in our understanding and warrant further investigation.

To address these questions, the current study employed a modified perceptual load Flanker task. This approach aimed to explore how emotional background and perceptual load interact in influencing attention processing. Participants were required to search for a target letter (H or S) and make discriminative responses among homogeneously (low load condition) or heterogeneously (high load condition) irrelevant letters in the central display, flanked by a congruent or incongruent interference letter presented at the left or right edges. Meanwhile, we used food and scene pictures to evoke high and low approach-motivated positive affect, and simple graphics and basic necessities pictures to induce neutral affect. With this manipulation, it was hypothesized that increasing approach motivation would facilitate target letter selection and reduce distractor letter interference, as seen in reaction times (RTs) and accuracy.

To further explore these interactions, the current study employed ERP technology, which offers high temporal resolution to precisely map the time course of attention processing. This approach allowed us to analyze whether perceptual load and emotional motivational intensity jointly influence relatively early or late attentional selection. According to literatures, early selective attention is primarily manifested in two ERP components in the parietal and occipital regions within 200 ms of stimulation. The P1 component, a positive wave occurring 80–120 ms post-stimulation, is recognized as the earliest attention-modulated sensory processing component. The N1 component, a negative wave emerging around 140–200 ms post-stimulation, is identified as an attention-regulated component involved in early visual discrimination (Mangun and Hillyard, 1990; Luck and Kappenman, 2012; Allon and Luria, 2019). The amplitudes of P1 and N1 increase when stimuli are attended to or receive greater attentional resources (Luck and Kappenman, 2012; Tunnermann et al., 2015). Additionally, the frontal and central N2 component, with a latency between 200 and 400 ms, reflects conflict monitoring or suppression of irrelevant stimuli. It is typically larger under incompatible conditions than compatible conditions (Heil et al., 2000; Veen and Carter, 2002a; Kanske and Kotz, 2010). Following the N2 component is the parietal P3 component, which occurs 300–600 ms after the stimulus and reflects the allocation of attentional resources. Difficult tasks or incongruent trials, which demand more attentional resources, typically elicit a smaller P3 amplitude (Kok, 2001; Wei and Zhou, 2020). ERP technology can determine whether the combined effect of perceptual load and emotional motivation occurs during the early or late stages of attention processing. If the motivational dimension of emotion or perceptual load influences the early stage of attention processing, the P1 and N1 amplitudes will be larger when stimuli are noticed or allocated more attentional resources. Based on previous studies, Flanker tasks under low approach-motivated positive affect or low perceptual load conditions are likely to evoke greater P1 or N1 amplitudes. However, if the interaction between emotional motivation and perceptual load affects later stages of attention processing, it is possible that high approach-motivated positive affect, high perceptual load conditions, and incongruent trials of the Flanker task may evoke a greater N2 amplitude and a smaller P3 amplitude compared to other conditions. Thus, the N2 and P3 components, which relate to the late selection of attention, will be selected for analysis.

## 2 Materials and methods

### 2.1 Participants

G* Power 3.1 (Faul et al., 2007) was used to calculate the required sample size, with an alpha level of 0.05, a power of 0.95, and a large effect size (f = 0.40). As the minimum number of participants required for the repeated-measures ANOVA was 15. Additionally, according to similar previous studies (e.g., Liu et al., 2016; Pandey and Gupta, 2022; Huang et al., 2023), a total of 26 university students (17 females and nine males; Mean age = 23, SD = 2.15) recruited from China participated in this study. All participants were right-handed and have normal or corrected-to-normal vision. All signed informed consent and were paid after the experiment. This study was approved by the Psychological Ethics Committee of the authors’ University.

### 2.2 Materials and experimental design

#### 2.2.1 Picture materials

A total of 30 pictures of neutral objects were selected from the International Affective Picture System (IAPS) (Lang et al., 1999), while 30 pictures of food and 30 pictures of scenery (natural landscapes, including mountains, grasslands, lakes, and so on) were selected from the Internet. Each image had a pixel dimension of 1,024 × 768. Three pictures of each type were used in the practice task, and 27 pictures were used in the formal experiment. After completing each block of the experiment, participants assessed their emotional responses to the pictures. The emotional self-assessment consisted of two parts: the first part consisted of 12 emotional adjectives (amusement, anger, anxiety, contentment, desire, disgust, engagement, fear, happiness, interest, sadness, and serenity) (Gable and Harmon-Jones, 2008). Among the 12 emotional adjectives, the adjective “Desire” was specifically related to approach motivation. The participants were not hungry when they were shown pictures of food. “Desire” indicates that participants were drawn to the images and had a stronger inclination to approach, get closer to, or acquire the content depicted in them. Participants assessed how they felt, using a scale ranging from 0 (no emotion) to 8 (strongest emotion). The second part was the Self-Assessment Manikin (SAM) (Bradley and Lang, 1994) to rate the valence of each picture (1 = very unpleasant, 9 = very pleasant) and its level of arousal (1 = calm, 9 = exciting).

#### 2.2.2 Flanker task stimulus

**FIGURE 1:**
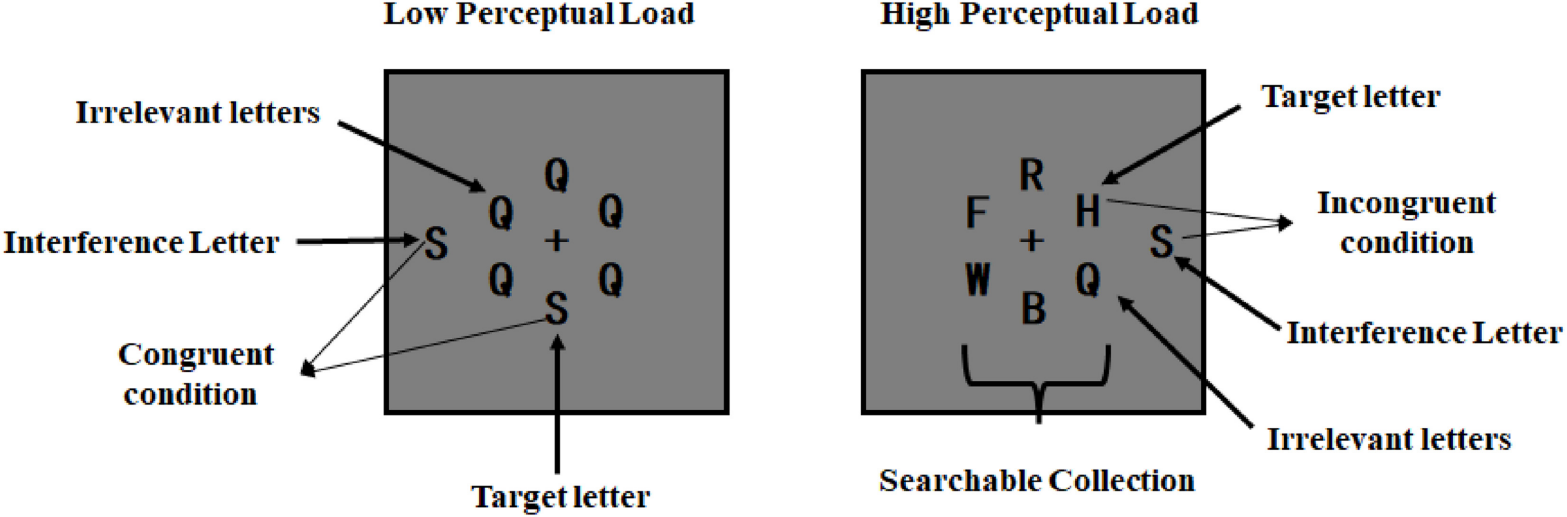
Sample stimuli of the Flanker task.

The Flanker task stimulus consisted of a centrally presented searchable collection of letters and an interference letter presented at the left or the right periphery (Figure 1). All letters were presented on a gray background. The viewing distance was 80 cm. Each letter was black and subtended 0.7° × 1° of the visual angles. The searchable collection included a target letter (H or S) and five irrelevant letters (selected from the following 12 letters: Q, W, Z, G, T, A, R, B, F, X, L, and K). The five irrelevant letters were the same in the low perceptual load condition, whereas different in the high perceptual load condition. The six letters were arranged in a virtual circle around the central fixation point. The interference letter was H or S, and the distance between interference letter and central fixation point of screen was 6.4°. The target letter and the interference letter were the same in the congruent condition, whereas different in the incongruent condition. This resulted in a 3 (Affect: neutral, high approach-motivated positive affect or low approach-motivated positive affect) × 2 (Perceptual load: high or low) × 2 (Congruency: congruent or incongruent) repeated measures design.

### 2.3 Procedure

**FIGURE 2:**
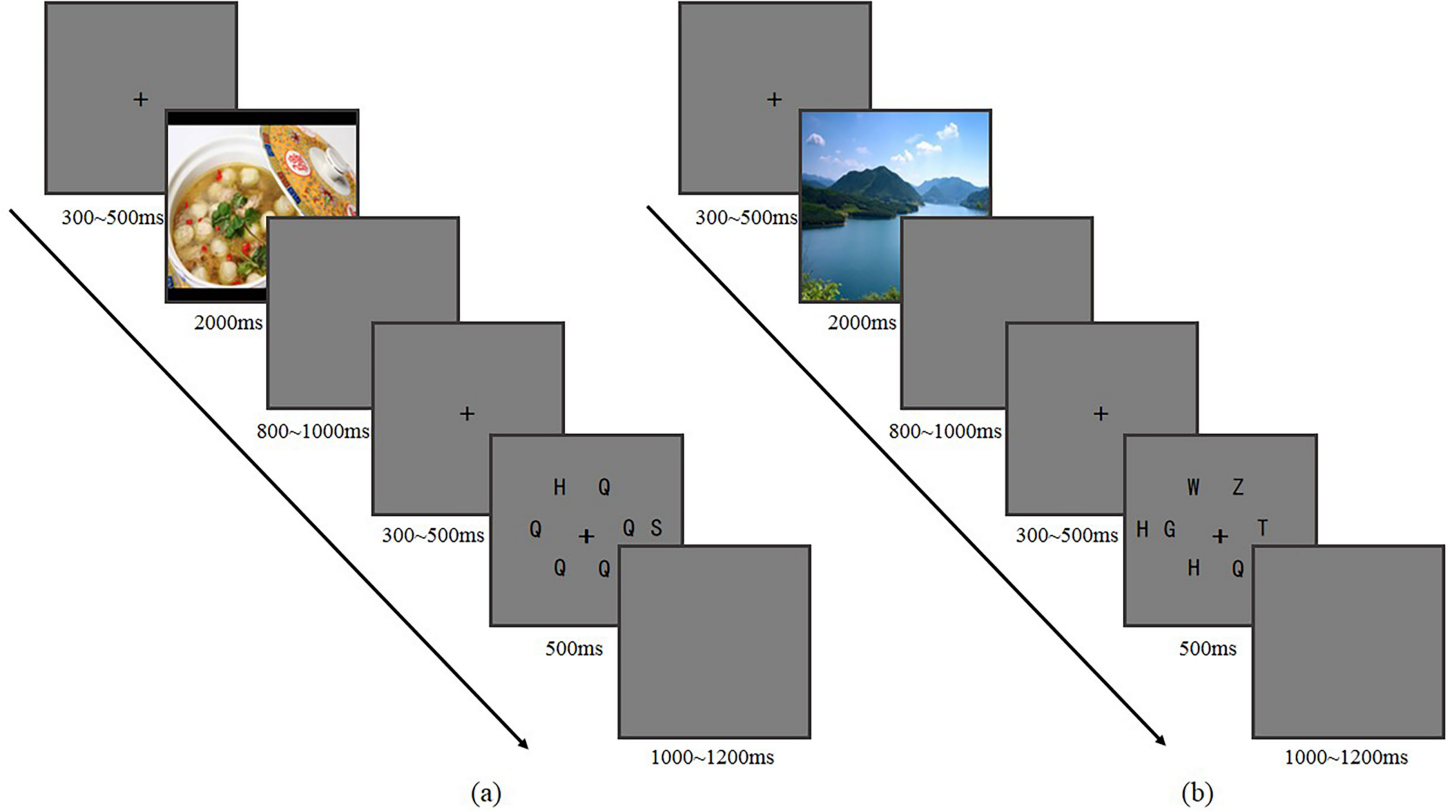
Procedure of the Flanker task [**(a)** shows an example of a incongruent trial in low perceptual load trials, and **(b)** shows an example of a congruent trial in high perceptual load trials].

The participants were asked to sit on the chair in an electromagnetically shielded and soundproofed room with soft light. All stimuli were presented on a 17-inch CRT display (refresh rate: 100 Hz). The present study adopted a block design with each participant completing three blocks: food, scene, and neutral object. The order of the blocks was counterbalanced using a Latin square design. Each block consisted of 216 trials. The ratio of high perceptual load and low perceptual load was each 50%, and the ratio of congruent and incongruent conditions was also each 50%. When combined in pairs, each of the four conditions accounted for one quarter. At the beginning of each trial, a central fixation cross “+” was presented for 300–500 ms, followed by a randomly selected affective or neutral picture for 2,000 ms. After that, a blank screen was present for 800–1,000 ms. Then a central fixation cross “+” was presented for 300–500 ms, and a randomly selected high or low perceptual load Flanker task was then displayed for 500 ms. At last, a blank screen was present for 1,000–1,200 ms (Figure 2). Participants were instructed to discriminate the target letter (H or S) by pressing numeric keypad with their index fingers. For half of the participants, the letter “H” was assigned to key 4 and “S” to key 6, operated by the left and right index fingers respectively. For the other half, “H” was assigned to key 6 and “S” to key 4, with the same finger-key mapping. Participants were only required to determine whether the target letter was H or S, without judging its position. In the experiment, the target letter had an equal probability of appearing to the left, right, top, or bottom of the central fixation point. The assignment of key to response hand was counterbalanced across participants. During the experiment, there were short breaks.

### 2.4 ERP recording and statistical analysis

Electroencephalograms (EEG) were recorded with 64 Ag/AgCl electrodes positioned in an elastic electrode cap at locations of the extended International 10–20 system by the Neuroscan system. The vertical electrooculograms (VEOGs) were recorded from electrodes placed above and below the left eye, and the horizontal electrooculograms (HEOGs) were monitored with electrodes at the outer canthi of both eyes, separately. The ground electrode is placed in the connection midpoint between the FPz electrode and the Fz electrode. A reference electrode was placed at the left ear mastoid and the reference voltage was changed offline to the average of left and right mastoid recordings. EEG signals were filtered with a bandpass of 0.05–40 Hz and sampled at a frequency of 500 Hz. The contact resistance between electrodes and scalp was kept below 5 kΩ. Scan 4.5 was used for ERP analysis. EOG blink artifacts were corrected using a linear regression estimate. False responses and the data with voltage exceeding ± 75 μV were excluded from analysis. Each averaging epoch lasted 900 ms from the flanker stimulus onset, including 100 ms prior to the flanker stimulus onset served as a baseline. The mean amplitudes of all correct response trials were overlapped and averaged separately. The mean numbers of epochs retained for each condition [12 conditions: Affect (3) × Perceptual load (2) × Congruency (2)] was 45 (rang: 37–54). The selection of electrode sites for the P1, N1, N2, and P3 components was based on prior studies with similar designs to the present study (Liu et al., 2016; Vogel and Luck, 2000; Luo et al., 2021; Huang et al., 2023). The waveforms of 88–108 ms at PO7 and PO8 electrodes was identified as P1, and the waveforms of 146–174 ms at PO7 and PO8 electrodes was identified as N1. The N2 component was identified as the average waveforms from 240 to 370 ms at the Fz, FCz, and Cz electrodes, and the P3 component was identified as the average waveforms from 400 to 600 ms at the Cz, CPz, and Pz electrodes. Repeated measures analyses of variance (ANOVAs) were performed on the behavior and EEG data. The Greenhouse-Geisser correction method was used. The degrees of freedom were uncorrected.

## 3 Results

### 3.1 Results on the effectiveness of high and low approach-motivated positive affect induction

At the end of each block, participants were asked to evaluate their emotion evoked by the pictures (Table 1). ANOVAs at three levels with Bonferroni correction were carried out for each self-assessment item. The results showed that the average scores of “anger,” “anxiety,” “disgust,” “fear,” and “sadness” were lower than 0.15. There was no significant main effect of picture type on these emotional adjectives (*p* = 0.773; *p* = 0.314; *p* = 0.522; *p* = 0.316; *p* = 0.419), indicating that these three types of pictures did not induce negative emotions. There were significant main effects of picture type on “amusement,” “contentment,” “desire,” “happiness,” “interest,” and “serenity” evaluation (*ps* < 0.001). Paired comparison results showed that the food pictures and scene pictures evoked more amusement, contentment, happiness, interest and serenity than neutral pictures (*ps* < 0.001), and there were no significant differences between food and scene pictures (*p* = 0.552; *p* = 0.799; *p* = 0.649; *p* = 0.518; *p* = 0.673). For the “desire” evaluation, there was a significant main effect [*F*(2, 50) = 222.88, *p* < 0.001, $\eta_{\text{p}}^{2}$ = 0.90]. Neutral pictures evoked significantly less desire than both food pictures and scene pictures (*ps* < 0.001). Moreover, food pictures evoked more desire than scene pictures (*p* = 0.008). This indicates that the approach-motivational intensity induced by food pictures was significantly higher than that induced by scene pictures. On the other hand, the evaluation results of valence and arousal showed significant main effects (*ps* < 0.001). The paired comparison results indicated that neutral pictures received significantly lower valence and arousal ratings compared to both food pictures and scenery pictures (*ps* < 0.001). However, no significant differences were observed between food pictures and scenery pictures in terms of valence (*p* = 1.00) and arousal (*p* = 1.00).

**TABLE 1:** Subjective affect self-assessment scores.

Self-assessment	Amusement	Anger	Anxiety	Contentment	Desire	Disgust	Serenity
Food pictures	4.19 (0.28)	0.04 (0.04)	0.19 (0.10)	5.92 (0.29)	7.08 (0.16)	0.08 (0.05)	4.89 (0.30)
Neutral pictures	1.81 (0.36)	0.08 (0.05)	0.15 (0.07)	1.77 (0.35)	1.46 (0.31)	0.15 (0.07)	4.12 (0.49)
Scene pictures	4.39 (0.39)	0.04 (0.04)	0.08 (0.05)	5.85 (0.22)	6.42 (0.21)	0.15 (0.07)	5.15 (0.25)
**Self-assessment**	**Fear**	**Happiness**	**Interest**	**Sadness**	**Engagement**	**Valence**	**Arousal**
Food pictures	0.04 (0.04)	6.27 (0.21)	6.77 (0.20)	0.08 (0.05)	6.12 (0.31)	6.92 (0.28)	5.54 (0.25)
Neutral pictures	0.15 (0.07)	2.50 (0.35)	2.15 (0.35)	0.15 (0.07)	2.81 (0.31)	4.62 (0.11)	3.46 (0.31)
Scene pictures	0.08 (0.05)	6.39 (0.24)	6.58 (0.24)	0.12 (0.06)	5.92 (0.24)	7.00 (0.25)	5.39 (0.22)

*Standard error in brackets.

The assessment results revealed that, compared to neutral object pictures, both food and scene pictures effectively induced positive affect. Additionally, the positive affect evoked by these two types of pictures only significantly differed in the dimension of “desire.” Specifically, food pictures effectively induced high approach-motivated positive affect, while scene pictures elicited low approach-motivated positive affect. This suggests that the key difference between the two types of positive affect only existed in the dimension of motivation.

### 3.2 Flanker task performance

The RTs and accuracies of Flanker tasks are shown in Tables 2, 3. First of all, an Affect (3) × Perceptual load (2) × Congruency (2) repeated measures ANOVA with Bonferroni correction for RTs data was performed. The results showed that the main effect of perceptual load was significant [*F*(1, 25) = 117.3, *p* < 0.001, $\eta_{\text{p}}^{2}$ = 0.82], which indicated that the RTs of high perceptual load trials (796.56 ± 107.55 ms) were significantly longer than those of low perceptual load trials (713.29 ± 81.85 ms). The main effect of congruency was significant, *F*(1, 25) = 24.61, *p* < 0.001, $\eta_{\text{p}}^{2}$ = 0.50, and this showed that the RTs of congruent trials (748.02 ± 94.20 ms) were significantly shorter than those of incongruent trials (761.83 ± 93.41 ms). No significant main effect of affect was found, *F*(2, 50) = 0.86, *p* = 0.43, $\eta_{\text{p}}^{2}$ = 0.03. However, the interaction of Affect × Perceptual load was significant, *F*(2, 50) = 15.36, *p* < 0.001, $\eta_{\text{p}}^{2}$ = 0.38, and the simple effect analysis showed that, only under the high perceptual load condition, the RTs of high approach-motivated positive affect trials (781.64 ± 101.66 ms) were significantly shorter than those of low approach-motivated positive affect trials (810.48 ± 121.28 ms) (*p* < 0.01). There were no significant interaction effect between congruency and affect *F*(2, 50) = 0.06, *p* = 0.94, $\eta_{\text{p}}^{2}$ = 0.38, no significant interaction effect between congruency and perceptual load *F*(1, 25) = 0.45, *p* = 0.51, $\eta_{\text{p}}^{2}$ = 0.02, and no significant three-factor interaction effect *F*(2, 50) = 2.53, *p* = 0.10, $\eta_{\text{p}}^{2}$ = 0.09.

**TABLE 2:** Mean (SE) reaction times (RTs) (ms) in response to Flanker task.

Conditions	Low perceptual load		High perceptual load	
	Congruent	Incongruent	Congruent	Incongruent
(Food pictures) high approach-motivated positive affect	710 (15.9)	717 (14.9)	773 (20.5)	790 (20.2)
(Scene pictures) low approach-motivated positive affect	700 (18.3)	716 (16.7)	804 (24.9)	816 (23.3)
(Neutral pictures) neutral affect	706 (19.1)	730 (19.9)	795 (21.1)	801 (22.7)

**TABLE 3:** Mean (SE) accuracies (%) in response to Flanker task.

Conditions	Low perceptual load		High perceptual load	
	Congruent	Incongruent	Congruent	Incongruent
(Food pictures) high approach-motivated positive affect	81.4 (3.9)	81.9 (3.8)	84.4 (3.2)	83.0 (3.3)
(Scene pictures) low approach-motivated positive affect	81.1 (4.5)	80.7 (4.4)	83.5 (2.7)	82.2 (2.8)
(Neutral pictures) neutral affect	83.8 (3.6)	81.4 (3.7)	84.2 (2.9)	82.5 (3.2)

Then, the results of Affect (3) × Perceptual load (2) × Congruency (2) repeated measures ANOVA with Bonferroni correction for accuracies data indicated that the main effect of congruency was significant, *F*(1, 25) = 10.77, *p* = 0.003, $\eta_{\text{p}}^{2}$ = 0.30, and this showed that the accuracies of congruent trials (0.83 ± 0.16) were significantly higher than those of incongruent trials (0.82 ± 0.16). No significant main effect of affect was found, *F*(2, 50) = 0.22, *p* = 0.74, $\eta_{\text{p}}^{2}$ = 0.01, and no significant main effect of perceptual load was found, *F*(1, 25) = 1.31, *p* = 0.26, $\eta_{\text{p}}^{2}$ = 0.05. There were no significant interaction effect between congruency and affect, *F*(2, 50) = 0.90, *p* = 0.40, $\eta_{\text{p}}^{2}$ = 0.04, no significant interaction effect between congruency and perceptual load, *F*(1, 25) = 0.58, *p* = 0.45, $\eta_{\text{p}}^{2}$ = 0.02, no significant interaction effect between affect and perceptual load, *F*(2, 50) = 0.36, *p* = 0.67, $\eta_{\text{p}}^{2}$ = 0.01, and no significant three-factor interaction effect, *F*(2, 50) = 0.53, *p* = 0.55, $\eta_{\text{p}}^{2}$ = 0.02.

### 3.3 ERPs results

**FIGURE 3:**
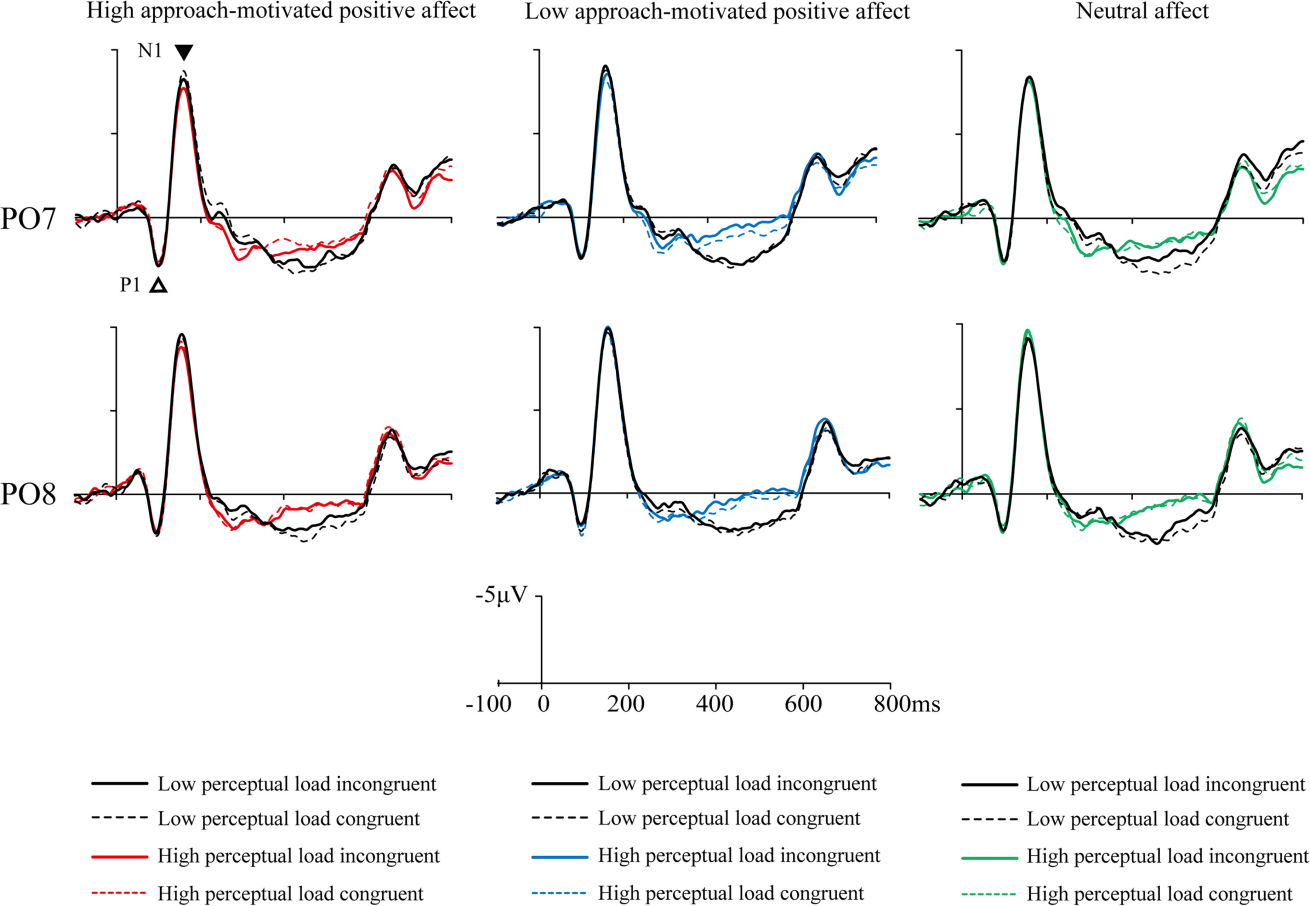
The grand mean event-related potentials (ERPs) at occipital-parietal electrodes PO7 and PO8 of the P1 and N1 induced by Flanker task under three emotional states.

**FIGURE 4:**
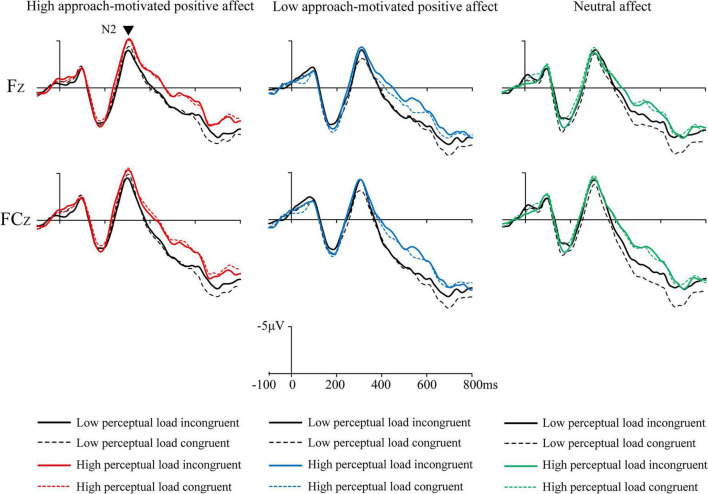
The grand mean event-related potentials (ERPs) at frontal electrodes Fz and FCz of the N2 induced by Flanker task under three emotional states.

**FIGURE 5:**
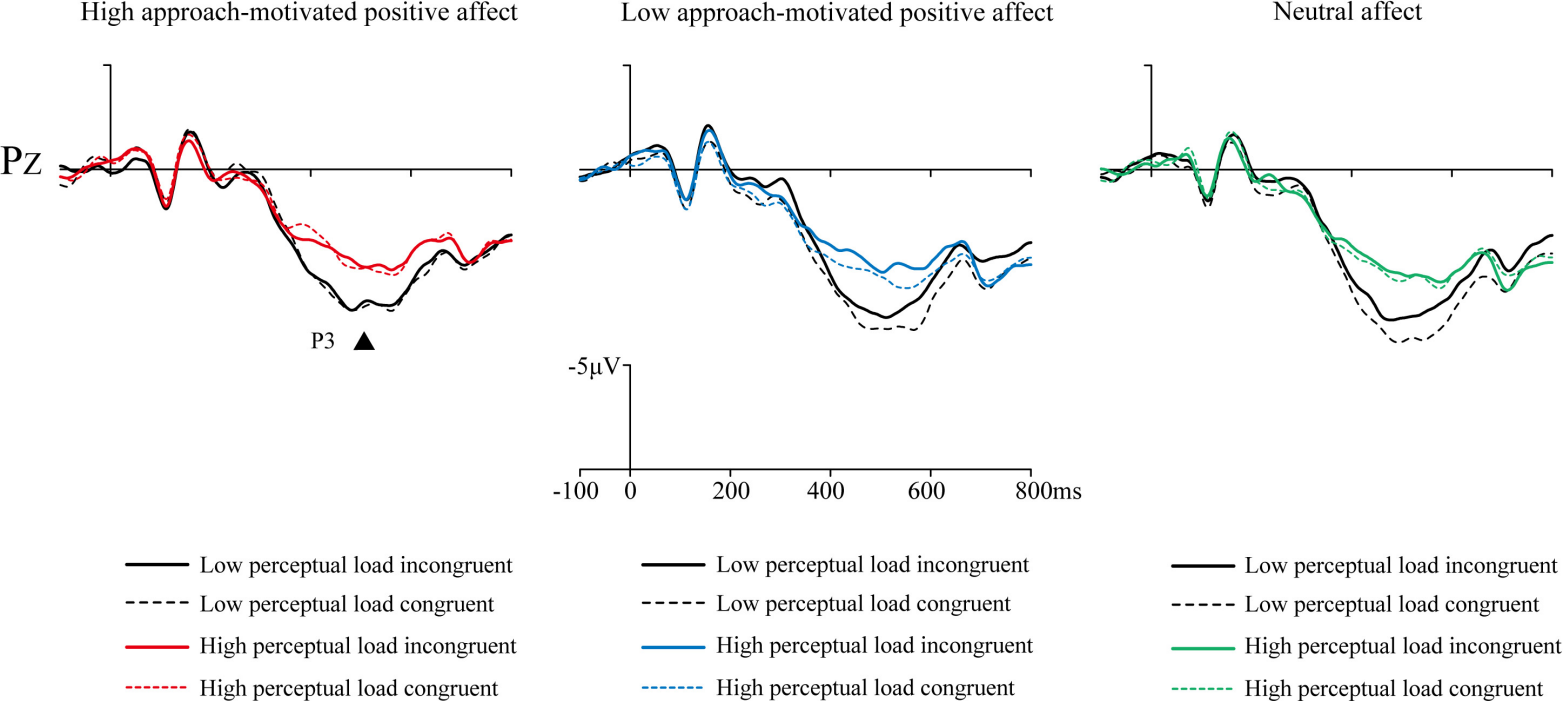
The grand mean event-related potentials (ERPs) at parietal electrode Pz of P3 induced by Flanker task under three emotional states.

Mean amplitudes of all correct response trials were computed at four latency intervals (N1: 88–108 ms, P1: 146–174 ms, N2: 240–370 ms, and P3: 400–600 ms) for each participant and condition type. The results are shown in Figures 3–5. Affect × Perceptual load × Congruency repeated-measures ANOVAs with Bonferroni correction were conducted on these intervals.

P1: The Affect (3) × Perceptual load (2) × Congruency (2) repeated measures ANOVA with Bonferroni correction for the average amplitude of PO7 electrode was performed. The results showed the main effects of perceptual load [*F*(1, 25) = 0.10, *p* = 0.76, $\eta_{\text{p}}^{2}$ = 0.004], affect [*F*(2, 50) = 1.54, *p* = 0.23, $\eta_{\text{p}}^{2}$ = 0.06], and congruency [*F*(1, 25) = 0.45, *p* = 0.51, $\eta_{\text{p}}^{2}$ = 0.02] were not significant. There were no significant interaction effect between affect and perceptual load [*F*(2, 50) = 0.01, *p* = 0.98, $\eta_{\text{p}}^{2}$ = 0], no significant interaction effect between affect and congruency [*F*(2, 50) = 0.52, *p* = 0.58, $\eta_{\text{p}}^{2}$ = 0.02], no significant interaction effect between perceptual load and congruency [*F*(1, 25) = 0.26, *p* = 0.61, $\eta_{\text{p}}^{2}$ = 0.01], and no significant three-factor interaction effect of affect, perceptual load, and congruency [*F*(2, 50) = 0.79, *p* = 0.43, $\eta_{\text{p}}^{2}$ = 0.03].

The Affect (3) × Perceptual load (2) × Congruency (2) repeated measures ANOVA with Bonferroni correction for the average amplitude of PO8 electrode was performed. The results showed the main effect of perceptual load [*F*(1, 25) = 0.004, *p* = 0.95, $\eta_{\text{p}}^{2}$ = 0], affect [*F*(1, 25) = 0.96, *p* = 0.39, $\eta_{\text{p}}^{2}$ = 0.04], and congruency [*F*(1, 25) = 0.93, *p* = 0.35, $\eta_{\text{p}}^{2}$ = 0.04] were not significant. There were no significant interaction effect between affect and perceptual load [*F*(2, 50) = 0.49, *p* = 0.60, $\eta_{\text{p}}^{2}$ = 0.02], no significant interaction effect between affect and congruency [*F*(2, 50) = 0.83, *p* = 0.44, $\eta_{\text{p}}^{2}$ = 0.03], no significant interaction effect between perceptual load and congruency [*F*(1, 25) = 0.45, *p* = 0.51, $\eta_{\text{p}}^{2}$ = 0.02], and no significant three-factor interaction effect of affect, perceptual load, and congruency [*F*(2, 50) = 0.24, *p* = 0.78, $\eta_{\text{p}}^{2}$ = 0.01].

N1: The Affect (3) × Perceptual load (2) × Congruency (2) repeated measures ANOVA with Bonferroni correction for the average amplitude of PO7 electrode was performed. The results showed the main effect of perceptual load was significant [*F*(1, 25) = 11.49, *p* = 0.002, $\eta_{\text{p}}^{2}$ = 0.32], the low perceptual load trials (−8.18 ± 5.09 μV) evoked a greater ERP than those of high perceptual load trials (−7.76 ± 5.16 μV). The main effect of affect [*F*(2, 50) = 1.61, *p* = 0.21, $\eta_{\text{p}}^{2}$ = 0.06] and congruency [*F*(1, 25) = 0.27, *p* = 0.61, $\eta_{\text{p}}^{2}$ = 0.01] were not significant. There were no significant interaction effect between affect and perceptual load [*F*(2, 50) = 0.74, *p* = 0.45, $\eta_{\text{p}}^{2}$ = 0.03], no significant interaction effect between affect and congruency [*F*(2, 50) = 2.40, *p* = 0.11, $\eta_{\text{p}}^{2}$ = 0.09], no significant interaction effect between perceptual load and congruency [*F*(1, 25) = 0.25, *p* = 0.62, $\eta_{\text{p}}^{2}$ = 0.01], and no significant three-factor interaction effect of affect, perceptual load, and congruency [*F*(2, 50) = 0.33, *p* = 0.70, $\eta_{\text{p}}^{2}$ = 0.01].

The Affect (3) × Perceptual load (2) × Congruency (2) repeated measures ANOVA with Bonferroni correction for the average amplitude of PO8 electrode was performed. The results showed the main effects of affect [*F*(2, 50) = 2.24, *p* = 0.13, $\eta_{\text{p}}^{2}$ = 0.08], perceptual load [*F*(2, 50) = 0.28, *p* = 0.60, $\eta_{\text{p}}^{2}$ = 0.01], and congruency [*F*(1, 25) = 0.66, *p* = 0.43, $\eta_{\text{p}}^{2}$ = 0.03] were not significant. The interaction effect between Affect and Perceptual load was significant, *F*(2, 50) = 3.36, *p* = 0.04, $\eta_{\text{p}}^{2}$ = 0.12, the simple effect analysis showed that, under the high perceptual load condition, the low approach-motivated positive affect (−9.22 ± 5.47 μV) evoked a greater ERP than high approach-motivated positive affect (−8.42 ± 5.06 μV; *p* = 0.039). There were no significant interaction effect between affect and congruency [*F*(2, 50) = 0.67, *p* = 0.50, $\eta_{\text{p}}^{2}$ = 0.03], no significant interaction effect between perceptual load and congruency [*F*(1, 25) = 0.01, *p* = 0.91, $\eta_{\text{p}}^{2}$ = 0.001], and no significant three-factor interaction effect of affect, perceptual load, and congruency [*F*(2, 50) = 0.76, *p* = 0.48, $\eta_{\text{p}}^{2}$ = 0.03].

N2: The Affect (3) × Perceptual load (2) × Congruency (2) repeated measures ANOVA with Bonferroni correction for the average amplitude of Fz, FCz, and Cz electrodes was performed. The results showed a significant main effect of affect, *F*(2, 50) = 5.62, *p* = 0.006, $\eta_{\text{p}}^{2}$ = 0.18, and the multiple comparison results showed that the high approach-motivated positive affect (−2.72 ± 2.88 μV) evoked a greater ERP than low approach-motivated positive affect (−1.72 ± 2.51 μV; *p* = 0.011), and the high approach-motivated positive affect tended to evoke a greater ERP than neutral affect (−2.02 ± 2.72 μV; *p* = 0.075). The ANOVA results also showed a significant main effect of perceptual load [*F*(1, 25) = 9.16, *p* = 0.006, $\eta_{\text{p}}^{2}$ = 0.27], and the high perceptual load trials (−2.48 ± 2.54 μV) evoked a greater ERP than the low perceptual load trials (−1.82 ± 2.68 μV). No significant main effect of congruency was found, *F*(1, 25) = 1.18, *p* = 0.29, $\eta_{\text{p}}^{2}$ = 0.05. There were no significant interaction effect between affect and perceptual load [*F*(2, 50) = 0.30, *p* = 0.73, $\eta_{\text{p}}^{2}$ = 0.01], no significant interaction effect between affect and congruency [*F*(2, 50) = 0.85, *p* = 0.42, $\eta_{\text{p}}^{2}$ = 0.03], no significant interaction effect between perceptual load and congruency [*F*(1, 25) = 0.73, *p* = 0.40, $\eta_{\text{p}}^{2}$ = 0.03], and no significant three-factor interaction effect of affect, perceptual load, and congruency [*F*(2, 50) = 1.21, *p* = 0.31, $\eta_{\text{p}}^{2}$ = 0.05].

P3: The Affect (3) × Perceptual load (2) × Congruency (2) repeated measures ANOVA with Bonferroni correction for the average amplitude of Cz, CPz, and Pz electrodes was performed. The results showed that the main effect of affect was significant [*F*(2, 50) = 4.49, *p* = 0.017, $\eta_{\text{p}}^{2}$ = 0.15]. The multiple comparison results showed that the neutral affect (6.02 ± 3.85 μV) evoked a greater ERP than high approach-motivated positive affect (4.99 ± 3.83 μV; *p* = 0.033), and the low approach-motivated positive affect (5.99 ± 4.05 μV) tended to evoke a greater ERP than high approach-motivated positive affect (4.99 ± 3.83 μV; *p* = 0.066). The main effect of perceptual load was significant [*F*(1, 25) = 69.63, *p* < 0.001, $\eta_{\text{p}}^{2}$ = 0.74], the low perceptual load trials (6.83 ± 4.07 μV) evoked a greater ERP than those of high perceptual load trials (4.50 ± 3.52 μV). The ANOVA results also showed a significant main effect of congruency [*F*(1, 25) = 4.81, *p* = 0.038, $\eta_{\text{p}}^{2}$ = 0.16], the flanker congruent trials (5.87 ± 3.81 μV) evoked a greater ERP than flanker incongruent trials (5.46 ± 3.73 μV). There were no significant interaction effect between affect and perceptual load [*F*(2, 50) = 0.19, *p* = 0.82, $\eta_{\text{p}}^{2}$ = 0.01], no significant interaction effect between affect and congruency [*F*(2, 50) = 0.67, *p* = 0.51, $\eta_{\text{p}}^{2}$ = 0.03], no significant interaction effect between perceptual load and congruency [*F*(1, 25) = 2.62, *p* = 0.12, $\eta_{\text{p}}^{2}$ = 0.10], and no significant three-factor interaction effect of affect, perceptual load, and congruency [*F*(2, 50) = 0.75, *p* = 0.46, $\eta_{\text{p}}^{2}$ = 0.03].

## 4 Discussion

The present study employed ERP technology and a modified perceptual load Flanker task to investigate how high and low approach-motivated positive affect interact with perceptual load to influence selective attention. Behavioral results indicated that congruent trials yielded higher accuracy than incongruent trials, and both congruency and perceptual load significantly affected reaction times (RTs). In incongruent trials, interference letters played a disruptive role, so that congruent trials resulted in shorter RTs than incongruent trials. As the perceptual load increased, task difficulty also rose, requiring more attentional resources for target letter identification. Consequently, high perceptual load tasks led to slower RTs than low load tasks (Lavie, 2010; Liu and Wang, 2014; Lü et al., 2007). Furthermore, a significant interaction between affect and perceptual load was observed. Under high perceptual load, individuals with high approach-motivated positive affect had shorter RTs than those with low approach-motivated positive affect. A plausible explanation is that individuals in high motivational emotional states may possess greater attentional resources or stronger cognitive control, enabling them to perform more efficiently in demanding tasks.

Event-related potential technology, which precisely reflects processing time, provided evidence of brain electrical activity to explain behavioral results. In studies of selective attention, a typical P1 component is elicited approximately 100 ms after stimulus onset (Luck and Kappenman, 2012), followed by an N1 component. Both components are distributed in the parietal-occipital lobes of the left and right cerebral hemispheres, such as the positions of the PO7 and PO8 electrodes (Marco-Pallarés et al., 2005; Seeck et al., 2017; Luo et al., 2021; Huang et al., 2023), and reflect the perception and classification of visual information (VanRullen and Thorpe, 2001; Vogel and Luck, 2000). The ERP results of this study showed no significant effect on P1, but revealed a significant main effect of perceptual load on N1 at the PO7 electrode. Specifically, low perceptual load trials elicited a greater N1 compared to high perceptual load trials. The N1 component is associated with the discriminative process applied to a restricted visual space (Vogel and Luck, 2000). Under low perceptual load conditions, individuals are able to perceive and discriminate all stimuli more rapidly, especially interference letters. Since low perceptual load trials are generally easier than high perceptual load trials, this condition may result in a greater N1 amplitude, indicative of more efficient discriminative processing. Additionally, a significant interaction effect between affect and perceptual load was observed at the PO8 electrode. Under the high perceptual load condition, individuals with low approach-motivated positive affect showed a greater N1 than individuals with high approach-motivated positive affect. The N1 component is known to reflect attentional allocation to stimuli (Mangun and Hillyard, 1990), with a greater N1 indicating a “gain control” mechanism of visual attention (Hillyard and Anllo-Vento, 1998; Hillyard et al., 1998). These electrophysiological results suggest that stimuli under low approach-motivated positive affect received more attentional resources or were discriminated more carefully. Analysis of the early Flanker task stages concluded that high and low approach-motivated positive affects impacted selective attention in a top-down manner under high perceptual load condition.

For the late stage of selective attention processing, the ERP analysis focused on how different levels of approach-motivated positive affect influenced response selection. Typically, the N2 component is generated in the cingulate gyrus and is distributed across the central frontal region (Veen and Carter, 2002a). Previous studies indicate that N2 reflects the resources required to detect and resolve conflicts, which is associated with the conflict monitoring function of the anterior cingulate cortex (ACC) (Heil et al., 2000; Kanske and Kotz, 2010; Veen and Carter, 2002b). The amplitude of the N2 component is also linked to the conscious recognition of target stimuli (Gehring and Knight, 2000). In this study, N2 analysis from 240 to 370 ms showed a significant main effect of perceptual load, with high perceptual load trials inducing a greater N2 compared to low perceptual load trials. This indicates that high perceptual load trials required more attentional monitoring and greater resources to identify and distinguish target letters within the centrally presented array. Additionally, a significant main effect of affect on N2 was found. Individuals with high approach-motivated positive affect showed a greater N2 than individuals with low approach-motivated positive affect. This implies that individuals under high approach-motivated positive affect exhibited stronger attention monitoring when searching for target letters. As a result, performance on the high perceptual load Flanker task was optimized under conditions of high approach-motivated positive affect.

Analysis of P3 from 400 to 600 ms showed that low perceptual load trials evoked a greater P3 than high perceptual load trials, and congruent trials evoked a greater P3 than incongruent trials. The P3 component, which is distributed in the central and parietal regions, serves as an effective indicator of inhibitory control (Yu et al., 2008; Yuan et al., 2008). Previous research has indicated that P3 amplitude is smaller in conditions requiring inhibition compared to those without inhibition (Markela-Lerenc et al., 2004; Qiu et al., 2006; Ramautar et al., 2006; Chen et al., 2008). Thus in incongruent trials, where target and interference letters conflict, the amplitude of P3 evoked by incongruent trials is smaller than in congruent trials due to the need to suppress interference letters. At the same time, the amplitude of P3 evoked by high perceptual load trials is smaller than in low perceptual load trials. Since P3 appears after the completion of stimulus evaluation (Kok, 2001; Polich, 2004; Wei and Zhou, 2020), differences in P3 also reflect higher-level information processing such as letter naming or identification. In high perceptual load trials, despite the presence of five different irrelevant letters in the searchable array not inducing incorrect responses to target letters, these irrelevant letters impair the recognition of target letters. The P3 analysis also revealed a significant main effect of affect. Specifically, the P3 amplitude under high approach-motivated positive affect was lower than that under low approach-motivated positive affect or neutral affect. Based on the above analysis, it could be inferred that individuals in high approach - motivated positive affect states exhibit stronger cognitive control against distractor letters or a higher - level ability in target letter identification.

In summary, this study aimed to investigate the effects of perceptual load and emotional motivation on attention selection. The behavioral results revealed a significant interaction between perceptual load and affect. Specifically, under high perceptual load conditions, reaction times (RTs) were significantly shorter for trials with high approach-motivated positive affect compared to those with low approach-motivated positive affect. ERP results suggest two possible reasons for this finding. First, the early ERP component N1 was influenced by the motivational dimension of positive affect. This indicates that the motivational dimension of emotion impacts selective attention in a top-down manner during the early stages of attention processing. Previous studies have found that low approach-motivated positive affect tends to broaden the scope of attention (Gable and Harmon-Jones, 2008, 2010a). This study demonstrated that low approach-motivated positive affect involves more extensive perception and discrimination processing of stimuli, which leads to slower reaction times in the late response stage. Therefore, under high perceptual load conditions, low approach-motivated positive affect prompts individuals to focus on a broader attentional range and discriminate interference letters, thereby prolonging reaction times compared to high approach-motivated positive affect. Moreover, the influence of the motivational dimension of positive affect on the late stage of selective attention has been confirmed. The analysis of the N2 and P3 components in the ERP results indicates that high approach-motivated positive affect enhances attention monitoring ability and allocates more attentional resources to inhibit interference compared to low approach-motivated positive affect. This heightened cognitive control facilitates the effective completion of the modified perceptual load Flanker task. Consequently, high approach-motivated positive affect promotes task completion, particularly for tasks with a certain level of difficulty. Under high perceptual load conditions, this effect is particularly pronounced, with RTs for high approach-motivated positive affect trials being significantly shorter than those for low approach-motivated positive affect trials.

In conclusion, this study highlights the facilitating role of high approach-motivated positive affect and the hindering effect of low approach-motivated positive affect on perceptual load Flanker tasks. Emotional context influences individual responses in a top-down manner, overshadowing the impact of perceptual load. These findings have significant theoretical implications for understanding how emotional information interacts with perceptual load in selective attention. Moreover, understanding the impact of high and low approach-motivated positive affect on cognitive tasks is crucial for optimizing performance in work and learning contexts.

## Data Availability

The original contributions presented in this study are included in this article/Supplementary material, further inquiries can be directed to the corresponding author.

## References

[B1] Allon A. S. Luria R. (2019). Filtering performance in visual working memory is improved by reducing early spatial attention to the distractors. *Psychophysiology* 56:e13323. 10.1111/psyp.13323 30609072

[B2] Bradley M. M. Lang P. J. (1994). Measuring emotion: The self-assessment manikin and the semantic differential. *J. Behav. Therapy Exp. Psychiatry* 25 49–59. 10.1016/0005-7916(94)90063-9 7962581

[B3] Cartwright-Finch U. Lavie N. (2007). The role of perceptual load in inattentional blindness. *Cognition* 102 321–340. 10.1016/j.cognition.2006.01.002 16480973

[B4] Chen A. Xu P. Wang Q. Luo Y. Yuan J. Yao D. (2008). The timing of cognitive control in partially incongruent categorization. *Hum. Brain Mapp.* 29 1028–1039. 10.1002/hbm.20449 17894393 PMC6871019

[B5] Faul F. Erdfelder E. Lang A. G. Buchner A. (2007). G*power 3: A flexible statistical power analysis program for the social, behavioral, and biomedical sciences. *Behav. Res. Methods* 39 175–191. 10.3758/bf03193146 17695343

[B6] Forster S. Lavie N. (2008a). Attentional capture by entirely irrelevant distractors. *Vis. Cogn.* 16 200–214. 10.1080/13506280701465049

[B7] Forster S. Lavie N. (2008b). Failures to ignore entirely irrelevant distractors: The role of load. *J. Exp. Psychol. Appl.* 14 73–83. 10.1037/1076-898X.14.1.73 18377168 PMC2672049

[B8] Fredrickson B. L. (2001). The role of positive emotions in positive psychology: The broaden-and-build theory of positive emotions. *Am. Psychol.* 56 218–226. 10.1037//0003-066x.56.3.218 11315248 PMC3122271

[B9] Gable P. A. Harmon-Jones E. (2008). Approach-motivated positive affect reduces breadth of attention. *Psychol. Sci.* 19 476–482. 10.1111/j.1467-9280.2008.02112.x 18466409

[B10] Gable P. A. Harmon-Jones E. (2010a). Late positive potential to appetitive stimuli and local attentional bias. *Emotion* 10 441–446. 10.1037/a0018425 20515232

[B11] Gable P. A. Harmon-Jones E. (2010b). The effect of low vs. High approach-motivated positive affect on memory for peripherally vs. Centrally presented information. *Emotion* 10 599–603. 10.1037/a0018426 20677877

[B12] Gehring W. J. Knight R. T. (2000). Prefrontal-cingulate interactions in action monitoring. *Nat. Neurosci.* 3 516–520. 10.1038/74899 10769394

[B13] Heil M. Osman A. Wiegelmann J. Rolke B. Hennighausen E. (2000). N200 in the eriksen-task: Inhibitory executive processes? *J. Psychophysiol.* 14 218–225. 10.1027/0269-8803.14.4.218

[B14] Hillyard S. A. Anllo-Vento L. (1998). Event-related brain potentials in the study of visual selective attention. *Proc. Natl. Acad. Sci. U S A.* 95 781–787. 10.1073/pnas.95.3.781 9448241 PMC33798

[B15] Hillyard S. A. Vogel E. K. Luck S. J. (1998). Sensory gain control (amplification) as a mechanism of selective attention: Electrophysiological and neuroimaging evidence. *Philos. Trans. R. Soc. B Biol. Sci.* 353 1257–1270. 10.1098/rstb.1998.0281 9770220 PMC1692341

[B16] Hu K. S. Bauer A. Padmala S. Pessoa L. (2012). Threat of bodily harm has opposing effects on cognition. *Emotion* 12 28–32. 10.1037/a0024345 21707143 PMC3207017

[B17] Huang J. Yang L. Li K. Li Y. Dai L. Wang T. (2023). Reduced attentional inhibition for peripheral distractors of angry faces under central perceptual load in deaf individuals: Evidence from an event-related potentials study. *Front. Hum. Neurosci.* 17:1162488. 10.3389/fnhum.2023.1162488 37662637 PMC10469715

[B18] Jefferies L. N. Enns J. T. Di Lollo V. (2019). The exogenous and endogenous control of attentional focusing. *Psychol. Res.* 83 989–1006. 10.1007/s00426-017-0918-y 28939935

[B19] Kanske P. Kotz S. A. (2010). Modulation of early conflict processing N200 responses to emotional words in a flanker task. *Neuropsychologia* 48 3661–3664. 10.1016/j.neuropsychologia.2010.07.021 20654636

[B20] Kok A. (2001). On the utility of P3 amplitude as a measure of processing capacity. *Psychophysiology* 38 557–577. 10.1017/s0048577201990559 11352145

[B21] Lang P. J. Bradley M. M. Cuthbert B. N. (1999). *International Affective Picture System (IAPS): Instruction Manual and Affective Ratings (Tech. Rep. No. A-4).* Gainesville, FL: The Center for Research in Psychophysiology, University of Florida.

[B22] Lavie N. (1995). Perceptual load as a necessary condition for selective attention. *J Exp. Psychol. Hum. Percept. Perform.* 21 451–468. 10.1037//0096-1523.21.3.451 7790827

[B23] Lavie N. (2005). Distracted and confused?: Selective attention under load. *Trends Cogn. Sci.* 9 75–82. 10.1016/j.tics.2004.12.004 15668100

[B24] Lavie N. (2010). Attention, distraction, and cognitive control under load. *Curr. Dir. Psychol.* 19 143–148. 10.1177/0963721410370295 26728138

[B25] Lavie N. Cox S. (1997). On the efficiency of attention selection: Efficient visual search results in inefficient rejection of distraction. *Psychol. Sci.* 8 395–398. 10.1007/s00426-001-0083-0 12132113

[B26] Lavie N. De Fockert J. W. (2003). Contrasting effects of sensory limits and capacity limits in visual selective attention. *Percept. Psychophys.* 65 202–212. 10.3758/BF03194795 12713239

[B27] Lavie N. Beck D. M. Konstantinou N. (2014). Blinded by the load: Attention, awareness and the role of perceptual load. *Philos. Trans. R. Soc.* 3699:20130205. 10.1098/rstb.2013.0205 24639578 PMC3965161

[B28] Lavie N. Hirst A. De Fockert J. W. Viding E. (2004). Load theory of selective attention and cognitive control. *J. Exp. Psychol. Gen.* 133 339–354. 10.1037/0096-3445.133.3.339 15355143

[B29] Liu F. Ding J. H. Zhang Q. (2016). Positive affect and selective attention: Approach-motivation intensity influences the early and late attention processing stages. *Acta Psychol. Sin.* 48 794–803. 10.3724/SP.J.1041.2016.00794 37113526

[B30] Liu Y. Wang Z. H. (2014). Positive affect and cognitive control: Approach-motivation intensity influences the balance between cognitive flexibility and stability. *Psychol. Sci.* 25 1116–1123. 10.1177/0956797614525213 24671575

[B31] Lü J. G. Wang L. Zhou X. L. (2007). Perceptual load, top-down attentional set and selective attention. *Psychol. Sci.* 3, 558–563. 10.1111/cdev.13604 34181274

[B32] Luck S. J. Kappenman E. S. (2012). “ERP components and selective attention,” in *The Oxford handbook of event-related potential components*, eds Luck S. J. Kappenman E. S. (New York: Oxford University Press), 295–327.

[B33] Luo X. Guo J. Li D. Liu L. Chen Y. Zhu Y. (2021). Atypical developmental trajectories of early perception among school-age children with attention deficit hyperactivity disorder during a visual search task. *Child Dev.* 92 e1186–e1197. 10.1111/cdev.13604 34181274

[B34] Mangun G. R. Hillyard S. A. (1990). Allocation of visual attention to spatial locations: Tradeoff functions for event-related brain potentials and detection performance. *Percept. Psychophys.* 47 532–550. 10.3758/bf03203106 2367174

[B35] Marco-Pallarés J. Grau C. Ruffini G. (2005). Combined ICA-LORETA analysis of mismatch negativity. *Neuroimage* 25 471–477. 10.1016/j.neuroimage.2004.11.028 15784426

[B36] Markela-Lerenc J. Ille N. Kaiser S. Fiedler P. Mundt C. Weisbrod M. (2004). Prefrontal-cingulate activation during executive con-trol: Which comes first? *Cogn. Brain Res.* 18 278–287. 10.1016/j.cogbrainres.2003.10.013 14741314

[B37] Pandey S. Gupta R. (2022). Irrelevant positive emotional information facilitates response inhibition only under a high perceptual load. *Sci. Rep.* 12:14591. 10.1038/s41598-022-17736-5 36028535 PMC9418248

[B38] Polich J. (2004). “Neuropsychology of P3a and P3b: A theoretical overview,” in *Brainwaves and mind: recent developments*, eds Moore N. Arikan K. (Wheaton, IL: Kjellberg), 15–29.

[B39] Qiu J. Luo Y. J. Wang Q. H. Zhang F. H. Zhang Q. L. (2006). Brain mechanism of stroop interference effect in Chinese characters. *Brain Res.* 1072 186–193. 10.1016/j.brainres.2005.12.029 16443198

[B40] Ramautar J. R. Kok A. Ridderinkhof K. R. (2006). Effects of stop-signal modality on the N2/P3 complex elicited in the stop-signal paradigm. *Biol. Psychol.* 72 96–109. 10.1016/j.biopsycho.2005.08.001 16157441

[B41] Seeck M. Koessler L. Bast T. Leijten F. Michel C. Baumgartner C. (2017). The standardized EEG electrode array of the IFCN. *Clin. Neurophysiol.* 128 2070–2077. 10.1016/j.clinph.2017.06.254 28778476

[B42] Tan J. Zhao Y. Wang L. Tian X. Cui Y. Yang Q. (2015). The competitive influences of perceptual load and working memory guidance on selective attention. *PLoS One* 10:e0129533. 10.1371/journal.pone.0129533 26098079 PMC4476695

[B43] Tunnermann J. Petersen A. Scharlau I. (2015). Does attention speed up processing? Decreases and increases of processing rates in visual prior entry. *J. Vision* 15 1–27. 10.1167/15.3.1 25733608

[B44] VanRullen R. Thorpe S. J. (2001). The time course of visual processing: From early perception to decision-making. *J. Cogn. Neurosci.* 13 454–461. 10.1162/08989290152001880 11388919

[B45] Veen V. Carter C. (2002a). The timing of action-monitoring processes in the anterior cingulate cortex. *J. Cogn. Neurosci.* 14 593–602. 10.1162/08989290260045837 12126500

[B46] Veen V. Carter C. (2002b). The anterior cingulate as a conflict monitor: fMRI and ERP studies. *Physiol. Behav.* 77 477–482. 10.1016/s0031-9384(02)00930-7 12526986

[B47] Vogel E. K. Luck S. J. (2000). The visual N1 component as an index of a discrimination process. *Psychophysiology* 37 190–203. 10.1111/1469-8986.3720190 10731769

[B48] Wei H. Zhou R. (2020). High working memory load impairs selective attention: EEG signatures. *Psychophysiology* 57:e13643. 10.1111/psyp.13643 32725929

[B49] Yu F. Yuan J. Luo Y. J. (2008). Auditory-induced emotion modulates processes of response inhibition: An event-related potential study. *Neuroreport* 20 25–30. 10.1097/WNR.0b013e32831ac9b1 18978645

[B50] Yuan J. He Y. Zhang Q. Chen A. Li H. (2008). Gender differences in behavioral inhibitory control: ERP evidence from a two-choice oddball task. *Psychophysiology* 45 986–993. 10.1111/j.1469-8986.2008.00693.x 18778319

